# Evaluation of the Effectiveness of Fermented Soybean-Lettuce Powder for Improving Menopausal Symptoms

**DOI:** 10.3390/nu14142878

**Published:** 2022-07-13

**Authors:** A Lum Han, Hee Kyung Lee, Hyun Soo Chon, Hyun Ock Pae, Min Sun Kim, Yong Il Shin, Sooah Kim

**Affiliations:** 1 Department of Family Medicine, Wonkwang University Hospital, Iksan 54538, Korea; yebbnkim@gmail.com; 2 HumanEnos LLC., Wanju 55347, Korea; 119bio@naver.com; 3 Department of Microbiology and Immunology, School of Medicine, Iksan 54538, Korea; hopae@wku.ac.kr; 4 Center for Nitric Oxide Metabolite at Wonkwang University, Iksan 54538, Korea; mskim@wku.ac.kr; 5 Department of Rehabilitation Medicine, Pusan National University School of Medicine, Busan 50612, Korea; rmshin01@gmail.com; 6 Department of Environment Science & Biotechnology, Jeonju University, Jeonju 55069, Korea; skim366@jj.ac.kr

**Keywords:** menopausal syndrome, soybean, lettuce, nitric oxide, fermentation, kupperman index

## Abstract

Menopause syndrome causes a decline in the quality of life of postmenopausal women. Hormone therapy is recommended for the treatment of menopausal syndromes. However, it has several side effects. Soybean has been safely used to relieve the symptoms of menopause. Lettuce has antidiabetic and anti-inflammatory effects and improves sleep quality. Natural nitric oxide metabolites are produced through fermentation, which increases the effectiveness of the functional substances. This study assessed the alleviation of menopausal syndrome symptoms by natural nitric oxide-containing soybean lettuce extract using the Kupperman index. This study included adult women with menopausal syndrome and a Kupperman index of ≥15. After a four-week study with 40 participants, the final analysis included 39 participants in the experimental group (*n* = 19) and the placebo group (*n* = 20). Body mass index, waist circumference, and the total cholesterol, low-density and high-density lipoprotein cholesterol, and triglyceride levels were not altered before and after treatment in both groups. There was a significant decrease in the Kupperman index after treatment in the experimental group, but no significant change was observed in the placebo group. Soybean lettuce extract alleviates menopause syndrome without any special side effects.

## 1. Introduction

As many women go through menopause, their ovarian function gradually declines over time, leading to changes in certain hormones and subsequent physical and psychological changes. Consequently, symptoms such as hot flashes, sweating, insomnia, atrophy of the genitourinary system, sleep disturbance, and anxiety appear, and the incidence of osteoporosis and cardiovascular diseases increases. These symptoms are individually different but are collectively known as menopause syndrome [1]. Hormone replacement therapy (HRT), mainly treated with estrogen and progesterone, is primarily used for the treatment of menopausal syndrome [1,2]. However, concerns regarding the side effects of HRT, such as cardiovascular disease, venous thromboembolism, or breast cancer, are increasing the interest in foods or extracts that are beneficial for menopausal symptoms [2].

Nitric oxide (NO) is a gas molecule with a very short half-life of three seconds. It plays an important role in maintaining homeostasis in the body by participating in vasodilation, neurotransmitters, and immune functions [3]. Abnormal NO production in the body affects the pathogenesis of various diseases such as cardiovascular disease, respiratory disease, platelet dysfunction, immune dysfunction, and sexual dysfunction [4]. NO synthetase synthesizes NO from l-arginine amino acids in the neurons, endothelial cells, and adipocytes. NO is oxidized to a very unstable gas and converted to nitrite ions (NO_2_^−^), which are then easily converted back to nitrate ions (NO_3_^−^). Conversely, NO_2_^−^ and NO_3_^−^ can be converted to NO through a reduction reaction as precursors to NO [5]. Therefore, NO_2_^−^ is a precursor or metabolite of NO.

NO can mediate the activation of Nrf2, which in turn triggers the antioxidant response element-driven transcription of phase 2 detoxification and antioxidant defense enzymes in the vascular cells. This may increase the relieving effects of isoflavones and lettuce extracts for menopausal syndrome [6]. In this study, improvement in the menopausal symptoms with the use of NO_2_^−^-containing soybean and lettuce extracts was assessed.

Soybeans contain 34–42% protein; 19–22% fat, such as linoleic acid, oleic acid, linolenic acid, and arachidonic acid; and 20% carbohydrates, such as sucrose. In addition, it contains approximately 1.5% phospholipids, such as 4–5% fiber, lecithin, and cephalin, and approximately 1% of substances, such as sterol, carotene, chlorophyll, and tocopherol. It also contains minerals, such as potassium, phosphorus, calcium, and vitamins, such as vitamin B1. Isoflavone, a soybean extract, is a phenolic compound based on C6–C3–C6 and has a structure similar to that of estrogen, a female hormone. It relieves various postmenopausal syndromes, lowers blood cholesterol, and prevents cardiovascular disease and osteoporosis. Thus, due to its female hormone-like action, it is also called a phytoestrogen. In addition, the physiological activity of isoflavones has been actively reported, showing a preventive effect on breast, prostate, ovarian, and colorectal cancers [7,8].

Lettuce extract has potential health effects, including inhibition of DNA damage, intracellular lipid peroxidation, pro-apoptotic pathways, and regulation of glucose metabolism. It has several medicinal properties, including antioxidant, anti-inflammatory, and analgesic activity [9]. Lettuce extract also has sedative-hypnotic properties [10]. Therefore, it may help relieve the symptoms of menopause.

The Kupperman index, used widely in Asia for the diagnosis of menopausal syndrome and in this study, was published by Kupperman et al. [11] as a standard. It is classified into six areas—vasomotor disorders, urinary symptoms, psychoneurological symptoms, motor symptoms, digestive symptoms, and systemic symptoms [11]. This index, consisting of 25 questions divided into six items, can be used to identify the degree and characteristics of menopausal disorders. A sum of the index points of 20 or less is classified as mild, 20–40 as moderate, and 40–60 as severe. A score of 60 or higher is considered within the critical range for menopausal syndrome [11].

A double-blind, randomized clinical trial was conducted with 60 postmenopausal women over 40 years of age with a Kupperman index score of ≥15 receiving placebo or red clover isoflavone supplementation (80 mg/day). Compared with placebo, red clover isoflavone supplementation in postmenopausal women significantly reduced the menopausal symptoms and had a positive effect on vaginal cytology and triglyceride levels [12]. However, no clinical studies have been conducted with mixed fermented soybean and lettuce extracts. Therefore, this study aimed to investigate the effect of fermented soybean and lettuce containing NO_2_− on menopausal syndrome.

## 2. Materials and Methods

### 2.1. Study Design

This study was a four-week randomized, double-blind clinical trial (registration number: KCT0007316). The participants underwent a screening test to evaluate whether the Kupperman index was 15 or higher and whether a clinical trial was possible. Patients received supplementation with soybean lettuce extract (SLE) and placebo pills if they were eligible to participate. The control group received a placebo under the same conditions as the experimental group, that is, the Kupperman index was 15 or higher, and the conditions were the same as the experimental group. After two weeks of pill supplementation, the participants visited the study center to check vital signs, side effects, and dosing adherence. Efficacy indicators, such as the Kupperman index, lipid profile, blood tests, and body weight, were assessed on the first and last days of the visit. Among the 40 participants enrolled, one dropped out. Therefore, 39 participants completed the study without any major violations.

The following methods were used to conduct a double-blind, randomized trial. Screening numbers were assigned to the participants who provided written informed consent to participate in the study. The judging number consisted of four digits, starting with an S followed by three digits (e.g., S001). The participants who passed the screening test were assigned a study subject number. The study participants were randomly assigned in sequence. The study subject numbers started with GCJ-R and were conventional with the last two digits. The numbers indicated the order of the study participants and ranged from one to 40. The number assigned to each subject was used as the subject identification code to identify the subject until the end of the trial. The participants were instructed to lead a normal life and avoid consuming other nutraceuticals or dietary supplements. They were instructed to avoid soy-based foods such as tofu and Cheonggukjang, nutritional supplements containing soy, lettuce in salads, and supplements containing lettuce extract.

### 2.2. Participants

Adult women aged 45–70 years who had reached menopause, were diagnosed with menopause syndrome, and had a Kupperman index of 15 or higher were included. Forty volunteers were recruited and randomly assigned to two groups. Patients who had undergone HRT within six weeks or had undergone surgical menopause; patients who were contraindicated for hormone therapy; patients with acute and severe cardiovascular diseases (heart failure, myocardial infarction, and stroke); those with serious liver function abnormalities; those with a history of clinically significant hypersensitivity to drugs and functional foods; those with a history of psychiatric treatment within two months prior to screening; and those with a history of drug or alcohol abuse were excluded. All 40 volunteers provided informed consent prior to treatment. The participants who attempted to discontinue pill supplementation by a physician were excluded.

### 2.3. Treatment and Pill Preparation

The participants received randomly assigned SLE or placebo tablets one at a time, four times a day (after each meal and before bedtime). Once a participant started taking the tablets, they were instructed to continue for a total of four weeks.

Two main test materials were used for this experiment: soybeans and lettuce. Soybeans were imported from Primorsky Krai, Russia, through Innofoodkorea (Incheon, Korea). Lettuce was purchased from a local farm in Wanju-gun, Korea, and the microorganism was produced by HumanEnos LLC (Wanju-gun, Korea).

#### 2.3.1. Manufacturing Process of Fermented Soybean and Lettuce Powder

Soybeans and lettuce were ground with distilled water at a ratio of 1:1. Subsequently, approximately 1% (1.0 × 108 cfu/mL) of GRAS-grade microorganisms was added to each of the materials. The temperature of the fermentation process was set at 30 °C, and the duration was 15 days for lettuce and 60 days for soybeans. Every factor that could affect the fermentation process, such as aeration, temperature, and pH, was managed throughout the process to maintain the optimal conditions for fermentation. The end product of the fermentation contained nitric oxide metabolites and antioxidants in a liquid form. After the fermentation, a centrifuge (1.5 t/Hr, disc separator, Alfatechkorea Corp. Gyeongsangbuk-do, Korea) was used to separate the supernatant, and the supernatant was condensed with an evaporator (1.5 t, Vacuum Evaporator, BDMPLANT, Gyeonggi-do, Korea), where the brix was at 4%. The condensed material was then frozen at −40 °C for 48 h and vacuum dried into powder form for 72 h with a freeze dryer (1.5 t, Vacuum Freeze Drier, Ajin E.S.R Co. Ltd., Daegu, Korea).

The fermented soybean and lettuce powder were then blended with a ratio of 8:2 and labeled as “Fermented Soybean/Lettuce Powder (HM-BL05).” The test and placebo capsules for the menopause clinical trial were produced by YUYU HealthCare CO., a GMP facility. The composition of the main and placebo pills used in this study is listed in Table 1.

#### 2.3.2. Nitrite Measurement

The following materials were used.

1. Sodium nitrite (NaNO_2_) stock solution, 100 mg/L in H_2_O
2. Standard solution, 10 mL stock solution in H_2_O total volume 1000 mL
3. Standard solution 0–10 mL in H_2_O, total volume 50 mL
4. Reagent 1: Sulfanilamide 0.5 g in 50% HCl (ratio 1:1), mix and elevate temperature, total volume 100 mL
5. Reagent 2: N-1-Naphthylethylenediamine dihydrochloride 10 g in H_2_O, total volume 100 mL

**Figure 1:**
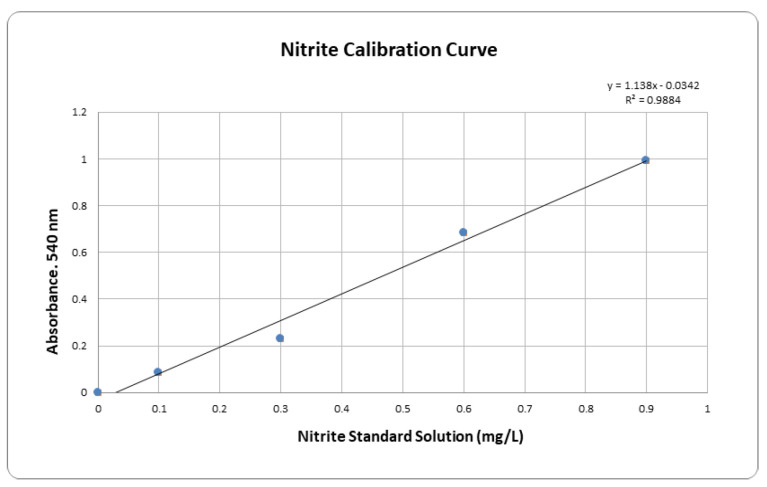
Standard curve of Nitrite.

The fermented aqueous solution of soybean and lettuce contained nitrite metabolites. A sample was screened for the presence of nitrite using a commercial kit (KnGlab, Seoul, Korea). In our modified Nitrite assay, Reagent 1 and Reagent 2 were added to a 50 mL dilute sample. This mixture was incubated at room temperature for 10 min. The absorption wavelength was 540 nm. The nitrite levels were calculated from nitrite standard curves generated in sodium nitrite standard solution. A typical standard curve is illustrated in Figure 1. Experimental data on the final fermentation product were provided as follows. Soybean and lettuce fermentation levels of nitrite, nitrate, chemical oxygen demand, and pH for the fermentation period were provided in Table 2. Soybean (after 59 days) and Lettuce (after 19 days) were fermented by Bacillus subtilis spp. to produce nitrites (Table 3).

### 2.4. Safety Assessment

The participants’ health was assessed through screening tests, including hematological examination, blood chemistry tests, and physical examination. Blood tests included the measurement of white and red blood cell counts and hemoglobin, hematocrit, platelet counts, total protein, albumin, alanine aminotransferase (ALT), aspartate aminotransferase (AST), blood urea nitrogen (BUN), and creatinine levels. The pulse rate and blood pressure were measured at each visit after a 10-min break. The participants were instructed to report side effects and medication compliance during supplementation.

### 2.5. Assessment of Menopausal Symptoms

The Kupperman index was used to rate the severity of 11 menopausal symptoms.

On this scale, menopause symptoms included hot flashes, night sweats, sleep difficulty, irritability, depression, dizziness, lack of concentration, joint pain, headache, palpitations, and vaginal dryness. Each symptom was rated according to its severity as follows: no symptom = 0, weak = 1, moderate = 2, and severe = 3. The total Kupperman index score was the sum of the individual symptom scores. A total score of 15 or higher was considered indicative of menopausal syndrome.

### 2.6. Hormone and Blood Chemistry Measurement

For blood tests, the participants fasted for >12 h before blood collection. The total cholesterol (TC), low-density lipoprotein cholesterol (LDL-C), high-density lipoprotein cholesterol (HDL-C), triglyceride (TG), non-HDL-cholesterol (Non-HDL-C), ALT, AST, gamma-glutamyl transferase, BUN, creatinine, glucose, high-sensitivity C-reactive protein, serum thyroid-stimulating hormone (TSH), estradiol, and follicle-stimulating hormone (FSH) levels were measured using a Hitachi 7600 automatic analyzer (Hitachi, Tokyo, Japan).

### 2.7. Statistical Analysis

All statistical analyses were performed using PASW Statistics 23 (previously SPSS Statistics) (SPSS version 23.0; IMP Corp., Armonk, NY, USA). All data obtained are expressed as the mean ± standard error or percentage (%) for categorical variables. The statistical significance was set at *p* < 0.05.

The sample size was determined to achieve 80% statistical power, with an alpha of 0.05. The sample size for each group was determined using a dropout rate of 20%. Efficacy parameters were analyzed in the per-protocol group, and safety parameters were analyzed in the intention-to-treat group. The chi-square test was performed to determine the baseline differences in the frequencies of the categorized variables between the groups. Student’s paired *t*-test was performed to assess the differences between the groups before and after the four-week intervention period.

## 3. Results

### 3.1. Participants

**Figure 2:**
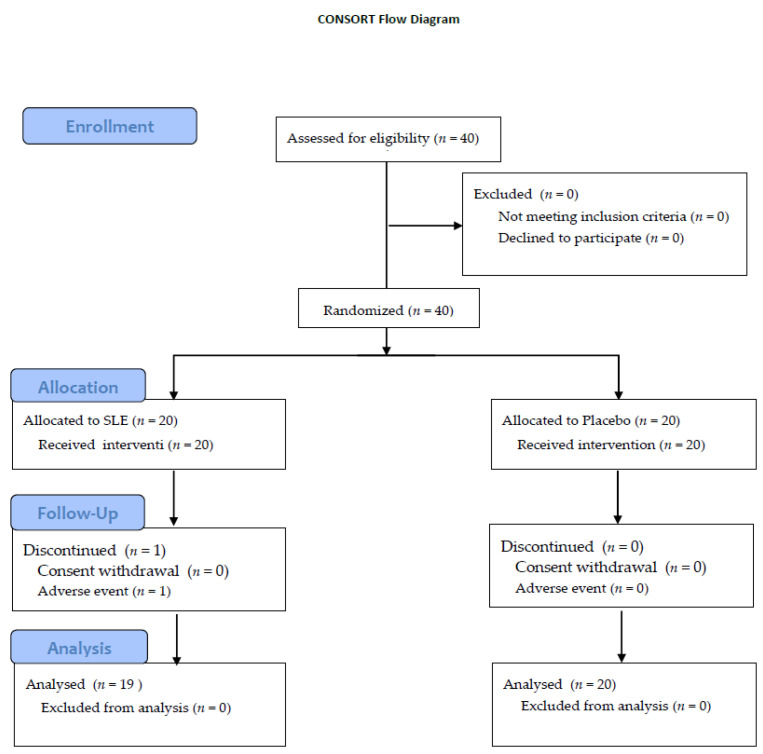
Flowchart of the inclusion and exclusion criteria for the participants.

Forty participants were enrolled and randomly assigned to SLE and placebo groups. Two weeks after taking the drug, one participant complained of indigestion and withdrew consent. All other participants completed the 4-week study. Thus, the final analysis included a total of 39 participants in the SLE (*n* = 19) and placebo (*n* = 20) groups (Figure 2).

### 3.2. Anthropometric Parameters

The general characteristics of the participants are shown in (Table 4). There were no significant differences in baseline characteristics, such as age, alcohol consumption, smoking, medical history, and exercise status, between the two groups.

Definitions: Smoking, individuals who are currently smoking (within two years); alcohol, individuals who currently intake alcohol more than once a month in the last year; Metabolic disease, individuals diagnosed with diabetes, hypertension, dyslipidemia, etc.

The values are presented as the mean ± standard deviation or number (percentage).

Cross-analysis was performed for alcohol intake, exercise, and disease, and ANOVA analysis of variance was performed for age.

### 3.3. Efficacy Assessment

There was no difference in WHR and BMI before and after treatment in the SLE and placebo groups. The Kupperman index showed no difference between the two groups before taking the pill; however, it showed a difference between the two groups after taking the pill, and the value decreased in the SLE group. There was no difference between the two groups in the changes in lipid profile and hormone levels, such as estradiol and FSH in blood tests; no difference was observed between the two groups even after treatment (Table 5).

There was a significant decrease in BMI after treatment in the placebo group. The decrease in the Kupperman index was statistically significant only in the SLE group. HbA1c decrease was statistically significant after treatment in both groups (Table 6).

## 4. Discussion

This study aimed to investigate the symptom improvement effects in menopausal syndrome after using SLE. SLE was prepared and used in this study to generate and immobilize NO metabolites that maintain NO_2_^−^, a NO metabolite, at a constant concentration for a long time. SLE containing NO_2_^−^ is expected to have a greater pharmacological effect. The results of this study showed that the difference in the mean of the Kupperman index before and after SLE administration was 7.48, which was a statistically significant difference. However, there was no statistically significant difference in the placebo-treated group.

Menopause is a biological process that can cause various symptoms, such as hot flashes and emotional changes, which many women struggle with [13]. In addition, it leads to osteoporosis and decreased metabolism, increasing the risk for various diseases. Vasomotor symptoms, such as hot flashes and sweating, are very common in the postmenopausal population and can cause physical and psychological discomfort [14]. E estrogen is still approved by the US Food and Drug Administration (FDA) as the most effective treatment for these menopausal symptoms [15]. However, a 2002 Women’s Health Initiative (WHI) study found that hormone replacement therapy increased the risk for breast cancer, stroke, and coronary heart disease in healthy postmenopausal women; therefore, the use of HRT has decreased [16]. Since the WHI data were first published in 2002, studies have suggested that an increased incidence of endometrial cancer may be associated with the decreased use of approved estrogen-progestogen therapy and increased use of compounded bioidentical HT [17]. Due to the serious side effects mentioned above, HRT is administered at the lowest effective dose for the shortest period of time to achieve the therapeutic goal [18]. The FDA recommends that women with menopausal syndrome should consider approved non-estrogen therapies prior to HRT [19]. Since then, postmenopausal women and health care professionals have been looking for an effective and safe alternative to HRT [14]. However, only a few clinical studies have studied the alleviation of menopausal symptoms with a substance that produces NO through fermentation of a mixture of isoflavones and lettuce extract. The role of NO was expected to maximize the efficacy of isoflavones and lettuce extracts in addition to antioxidant and anti-inflammatory actions. A previous study showed that the antihypertensive effect of garlic was increased by NO generated during fermentation [20]. Another previous study has investigated the anti-diabetic effect of noodles containing fermented lettuce extract in diabetic mice. Noodles with fermented lettuce extract were high in γ-aminobutyric acid content, antioxidant capacity, and total polyphenol content, demonstrating that mice fed this noodle had lower blood sugar levels and insulin resistance than mice fed standard noodles [21]. This study suggested the antioxidant role of fermented lettuce extract in menopausal syndrome.

Isoflavones, compounds that are abundant in soybeans, play a role in relieving menopausal symptoms by exerting estrogen-like effects. There are two types of estrogen receptors: ERα, the predominant form in the breast and uterus, and ERβ, the predominant form in the cardiovascular, genitourinary, and skeletal systems [22]. Isoflavones bind weakly to Erα and stronger to Erβ. Therefore, isoflavones can be expected to relieve the symptoms of menopausal syndrome via ERb binding.

As menopausal symptoms are subjective, the placebo effect is unavoidable. One study showed a reduction in hot flashes in all patients consuming isoflavone-rich soy, isoflavone-deficient soy, or whey protein [23]. Another study found no difference in the frequency of menopausal symptoms after 12 weeks of treatment with isoflavones or placebo [23].

Nevertheless, more recent studies favor the use of isoflavones to treat menopausal symptoms such as hot flashes [24]. One study reported a decrease in the Kupperman index in postmenopausal women after six months of receiving a herbal supplement containing 72 mg of isoflavones from soy and red clover [25]. In another small prospective study of 51 women who were administered 60 mg of isoflavones daily for 12 weeks to relieve menopausal syndrome, hot flashes and night sweats were reduced by 57% and 43%, respectively. The treatment did not change the levels of circulating estradiol or follicle-stimulating hormone [26].

A significant number of women experience sleep disturbances after menopause, with 26% reporting severe symptoms that affect daytime functioning. The severity of sleep dissatisfaction in postmenopausal women has been reported, and there may be an association between hot flashes, sleep deprivation, and depression [27].

Lettuce is one of the most widely consumed vegetables worldwide. However, its nutritional value is underestimated. Lettuce is also a good source of various other bioactive compounds. In vitro and in vivo studies have shown the anti-inflammatory, cholesterol-lowering, and anti-diabetic activities of the bioactive compounds in lettuce [9].

Traditionally, lettuce is recommended for its hypnotic effects. One study investigated the sleep-prolonging effects of lettuce. In a mouse experiment in which sleep was induced by pentobarbital administration, lettuce administration increased sleep duration and reduced sleep latency [10].

In a prospective randomized clinical trial, 100 pregnant women with insomnia aged 20–45 years were administered capsules or placebos containing 1000 mg of lettuce seeds daily for two weeks. Lettuce seeds were found to reduce insomnia during pregnancy and may be recommended for the treatment of pregnancy-related insomnia [28].

The Kupperman index is a standard widely used in Asia for diagnosing menopausal syndrome and is divided into six categories—vasomotor disorders, urinary symptoms, psychoneurological symptoms, motor symptoms, digestive symptoms, and systemic symptoms. It is an index that identifies the degree and characteristics of disability [11].

The natural NO used in the product in this study is stabilized through fermentation and aging based on bioconversion technology using natural materials. It stabilizes NO from natural materials and has the functionality of natural materials. Compared with normal NO_3_^−^, natural NO and its metabolites from fermentation have the advantage of directly supplying nitric oxide to the body and having fewer side effects even when overdosed [29,30].

A major limitation of our study is the small sample size, which limits the generalization of the results to other populations of menopausal individuals. In addition, the participants did not use the product for a long duration. In addition, since we relied on participant-reported data, we did not control for all factors that could influence menopausal symptoms. Nevertheless, this study is meaningful as it is the first study to test the improvement of symptoms of menopausal syndrome with soybean-lettuce mixture extract. It would also be valuable to confirm the efficacy and effect of natural nitrogen obtained through the fermentation process.

## 5. Conclusions

This study was a randomized, double-blind clinical trial conducted to assess the effect of the soybean-lettuce mixture extract on the improvement of menopausal syndrome. SLE contained natural NO that was stabilized through the fermentation process; therefore, its functionality was increased. After four weeks of treatment, symptom improvement in participants with menopause syndrome was confirmed using the Kupperman index. Although the period of administration was not long, this study is meaningful as it is the first study to verify the effect of SLE containing NO on menopausal syndrome. In the future, a better-designed study with more participants and an extended period of use is needed to verify our findings. 

## Figures and Tables

**Table 1:** Composition of pills.

Ingredient	Content (mg)	
	Experiment Pill	Placebo Pill
Fermented Soybean/Lettuce Powder (HM-BL05)	350	80
crystalline cellulose	0	270
corn starch	110	110
Bamboo Sap Extract Powder	10	10
Cottonseed Oil Powder	10	10
dry yeast	20	20
Total	500	500

**Table 2:** Experimental data.

Contents	Nitrite (mg/L)		Nitrate (mg/L)		Chemical Oxygen Demand (mg/L)		PH	
	Fermentation							
	Before (0 day)	After (59 day)	Before (0 day)	After (59 day)	Before (0 day)	After (59 day)	Before (0 day)	After (59 day)
Soybean	N.D	276	324	145	3600	780	4.44	8.66
Lettuce	N.D	159.3	4527	230	1450	340	4.5	7.6

**Table 3:** Nitrite production after fermentation.

Sample	Abs (540 nm)	Dilution Factor (Fold)	Nitrite (mg/L)
Soybean fermented solution	0.483	2000	276
Lettuce fermented solution	0.264	2000	159.3

**Table 4:** General characteristics of the participants.

Value	Group (*n* = 39)		*p*
	SLE (*n* = 19)	Placebo (*n* = 20)	
Age (years)	53.63 ± 4.66	53.05 ± 5.62	0.728
Drinking (%)	1(5.3)	1 (5.0)	0.970
Smoking (%)	0	0	-
Exercise (%)	7(36.8)	3 (15.0)	0.118
Metabolic disease (%)	2(10.5)	2 (10.0)	0.957
Total (%)	19	20	0.728

Abbreviations: SLE, soybean lettuce extract.

**Table 5:** Differences between groups before and after treatment.

Value	Before			After		
	SLE	Placebo	*p*	SLE	Placebo	*p*
SBP (mm Hg)	128.21 ± 18.08	131.2 ± 14.33	0.570	129.47 ± 15.09	132.55 ± 14.35	0.518
DBP (mm Hg)	75.05 ± 11.69	78.45 ± 9.04	0.315	74.32 ± 11.6	79.25 ± 9.08	0.146
WHR	86.89 ± 6.06	87 ± 8.52	0.965	86 ± 7.47	86.9 ± 8.24	0.723
BMI	24.41 ± 4.27	25.63 ± 3.46	0.334	24.47 ± 4.26	25.4 ± 3.53	0.466
Kupperman index	27.16 ± 6.52	26.45 ± 6.45	0.735	19.68 ± 5.09	25.4 ± 5.63	0.002
TSH (uIU/mL)	2.28 ± 1.73	2.64 ± 1.63	0.505	2.43 ± 1.72	2.24 ± 1.44	0.708
Free T4 (ng/dL)	1.2 ± 0.21	1.13 ± 0.11	0.176	1.22 ± 0.18	1.15 ± 0.09	0.123
Estradiol (pg/mL)	64.64 ± 208.47	18.18 ± 37.62	0.333	16.75 ± 44.91	63.99 ± 205.87	0.335
FSH (mIU/mL)	57.58 ± 28.68	60.78 ± 35.96	0.762	61.04 ± 28.37	55.39 ± 34.64	0.582
Glucose (mg/dL)	115.42 ± 53.17	102.25 ± 10.94	0.285	114.32 ± 45.86	101.65 ± 9.13	0.234
AST (IU/L)	21.21 ± 3.79	27.7 ± 16.87	0.110	21.74 ± 5.91	26.5 ± 13.56	0.167
ALT (IU/L)	18.42 ± 8.21	23.7 ± 22.66	0.345	20.74 ± 12.51	23.05 ± 23.58	0.706
GGT(IU/L)	21.16 ± 11.24	17.7 ± 8.03	0.274	22.95 ± 14.85	16.95 ± 8.13	0.124
BUN (mg/dL)	12.21 ± 2.86	13.19 ± 1.87	0.211	13.42 ± 2.97	11.87 ± 2.4	0.080
Cr (mg/dL)	0.67 ± 0.08	0.67 ± 0.06	0.973	0.68 ± 0.1	0.7 ± 0.07	0.510
Uric Acid (mg/dL)	4.59 ± 0.84	4.64 ± 0.76	0.859	4.71 ± 1.16	4.85 ± 0.92	0.679
TC (mg/dL)	206.53 ± 34.96	217.7 ± 42.79	0.379	217.42 ± 44.44	209 ± 50.08	0.583
Triglyceride (mg/dL)	122.68 ± 56.72	148.15 ± 140.9	0.468	158.47 ± 138.53	108.75 ± 61.15	0.152
HDL-C (mg/dL)	58.84 ± 19.9	59.7 ± 13.42	0.875	58.95 ± 21.97	59.85 ± 10.89	0.871
LDL-C (mg/dL)	117.79 ± 31.93	129.85 ± 44.11	0.337	124.74 ± 42.25	123.85 ± 46.92	0.951
hsCRP (mg/dL)	1.16 ± 0.74	0.93 ± 0.47	0.246	1.22 ± 0.85	1.29 ± 1.03	0.822
HbA1c (%)	5.68 ± 1.09	5.5 ± 0.4	0.471	5.57 ± 1.12	5.42 ± 0.45	0.574

Abbreviations: SBP, systolic blood pressure; DBP, diastolic blood pressure; WHR, waist-hip ratio; BMI, body mass index; TSH, thyroid-stimulating hormone; Free T4, Free Thyroxine 4; FSH, folli-cle-stimulating hormone ALT, alanine aminotransferase; AST, aspartate aminotransferase; BUN, blood urea nitrogen; TC, total cholesterol; LDL-C, low-density lipoprotein cholesterol; HDL-C, high-density lipoprotein cholesterol; TG, triglyceride; GGT, gamma-glutamyl transferase; hsCRP, high-sensitivity C-reactive protein; TSH, thyroid-stimulating hormone; hsCRP, high-sensitivity C-reactive protein; HbA1c, Hemoglobin A1C. The values are presented as the mean ± standard deviation. A paired t-test was performed to analyze the statistical significance of each variable before and after administration.

**Table 6:** Efficacy evaluation between the groups before and after treatment.

Value	SLE Group			Placebo Group		
	Before	After	*p*	Before	After	*p*
SBP (mm Hg)	128.21 ± 18.08	129.47 ± 15.09	0.605	131.2 ± 14.33	132.55 ± 14.35	0.644
DBP (mm Hg)	75.05 ± 11.69	74.32 ± 11.6	0.598	78.45 ± 9.04	79.25 ± 9.08	0.697
WHR	86.89 ± 6.06	86 ± 7.47	0.287	87 ± 8.52	86.9 ± 8.24	0.789
BMI	24.41 ± 4.27	24.47 ± 4.26	0.468	25.63 ± 3.46	25.4 ± 3.53	0.027
Kupperman index	27.16 ± 6.52	19.68 ± 5.09	<0.0001	26.45 ± 6.45	25.4 ± 5.63	0.292
TSH (uIU/mL)	2.28 ± 1.73	2.43 ± 1.72	0.519	2.64 ± 1.63	2.24 ± 1.44	0.156
Free T4 (ng/dL)	1.2 ± 0.21	1.22 ± 0.18	0.383	1.13 ± 0.11	1.15 ± 0.09	0.466
Estradiol (pg/mL)	64.64 ± 208.47	16.75 ± 44.91	0.249	18.18 ± 37.62	63.99 ± 205.87	0.173
FSH (mIU/mL)	57.58 ± 28.68	61.04 ± 28.37	0.505	60.78 ± 35.96	55.39 ± 34.64	0.074
Glucose (mg/dL)	115.42 ± 53.17	114.32 ± 45.86	0.793	102.25 ± 10.94	101.65 ± 9.13	0.513
AST (IU/L)	21.21 ± 3.79	21.74 ± 5.91	0.981	27.7 ± 16.87	26.5 ± 13.56	0.406
ALT (IU/L)	18.42 ± 8.21	20.74 ± 12.51	0.310	23.7 ± 22.66	23.05 ± 23.58	0.294
GGT(IU/L)	21.16 ± 11.24	22.95 ± 14.85	0.265	17.7 ± 8.03	16.95 ± 8.13	0.159
BUN (mg/dL)	12.21 ± 2.86	13.42 ± 2.97	0.132	13.19 ± 1.87	11.87 ± 2.4	0.038
Cr (mg/dL)	0.67 ± 0.08	0.68 ± 0.1	0.354	0.67 ± 0.06	0.7 ± 0.07	0.003
Uric Acid (mg/dL)	4.59 ± 0.84	4.71 ± 1.16	0.482	4.64 ± 0.76	4.85 ± 0.92	0.238
TC (mg/dL)	206.53 ± 34.96	217.42 ± 44.44	0.097	217.7 ± 42.79	209 ± 50.08	0.113
Triglyceride (mg/dL)	122.68 ± 56.72	158.47 ± 138.53	0.643	148.15 ± 140.9	108.75 ± 61.15	0.218
HDL-C (mg/dL)	58.84 ± 19.9	58.95 ± 21.97	0.809	59.7 ± 13.42	59.85 ± 10.89	0.213
LDL-C (mg/dL)	117.79 ± 31.93	124.74 ± 42.25	0.181	129.85 ± 44.11	123.85 ± 46.92	0.163
hsCRP (mg/dL)	1.16 ± 0.74	1.22 ± 0.85	0.643	0.93 ± 0.47	1.29 ± 1.03	0.050
HbA1c (%)	5.68 ± 1.09	5.57 ± 1.12	0.033	5.5 ± 0.4	5.42 ± 0.45	0.008

Abbreviations: SBP, systolic blood pressure; DBP, diastolic blood pressure; WHR, waist-hip ratio; BMI, body mass index; TSH, thyroid-stimulating hormone; Free T4, Free Thyroxine 4; FSH, follicle-stimulating hormone ALT, alanine aminotransferase; AST, aspartate aminotransferase; BUN, blood urea nitrogen; TC, total cholesterol; LDL-C, low-density lipoprotein cholesterol; HDL-C, high-density lipoprotein cholesterol; TG, triglyceride; GGT, gamma-glutamyl transferase; hsCRP, high-sensitivity C-reactive protein; TSH, thyroid-stimulating hormone; hsCRP, high-sensitivity C-reactive protein; HbA1c, Hemoglobin A1C. The values are presented as the mean ± standard deviation. Paired *t*-test and Wilcoxon signed rank test were performed to analyze the statistical significance of each variable before and after administration.

## Data Availability

Not applicable.
